# Supplementary material for: Quantifying Loopy Network Architectures
Source: PLoS One. 2012 Jun 6;7(6):e37994. doi: 10.1371/journal.pone.0037994 (PMC3368948; doi:10.1371/journal.pone.0037994)
Supplement: Supporting Information S1 — (PDF) [file pone.0037994.s001.pdf]

Supporting Information for the article "Quantifying loopy network architectures"

Eleni Katifori Marcelo O. Magnasco

1 Analytical calculation of the Asymmetry

The Asymmetry of certain models can be calculated analytically in closed form. For example, the Asymmetry (Eq. (2) of main text) of the infinite gradient model can be calculated by considering that the nestedness model is purely additive, with $s_j = d(j) - 1$ and $r_j = 1$, where $d(j)$ is the degree of the node j. The associated nesting tree of such a model is shown in Fig. S1(a) and the Asymmetry $Q_T(t_n)$ reads:

$$Q_T(t_n) = \frac{1}{w(t_n)} \sum_{j=1}^{d(n)-1} w_j \frac{d(j)-2}{d(j)-1} \quad (1)$$

Using the weight function:

$$w_j = d(j) - 1, \quad (2)$$

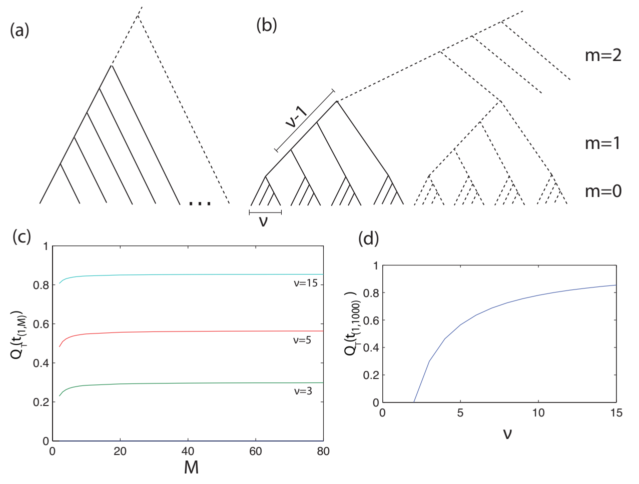

Figure 1: (a) Purely additive nesting tree. (b) Self-similar tree, with an additive building block of ν elements. (c) Asymmetry $Q_0(t_{(1,M)})$ as a function of the maximum network order M. (d) Asymmetry of self similar models as a function of the building block size ν.

it is trivial to show that:

$$Q_T(t_n) = 1 - \frac{2}{d(n)}. \quad (3)$$

The Asymmetry of a self similar model with an additive building block that consists of ν elements can similarly be calculated in closed form. This model is constructed as follows. At iteration order $m = M$, the graph consists of just the additive building block (see e.g. Fig. 2(d)(ii)) of the main text). At iteration order $m = M - 1$, the graph is augmented by adding the additive building block, with appropriately rescaled link weights, inside each of the ultimate loops of the order $m = M$ graph. We continue this nesting procedure until $m = 0$. It should be noted that the graph at $m = M$ does not need to be the complete building block, just a subgraph of the original building block.

The nesting tree of such a model is shown in Fig. S1(b). If m is the iteration order of node J, then it is easy to see that $s_J = d(J) - \nu^m$ and $r_J = \nu^m$. If we now define $j \equiv d(J)/\nu^m - 1$, then the partition asymmetry simply reads:

$$q(r_J, s_J) = \frac{j-1}{j}. \quad (4)$$

Any node J of the nesting tree can be characterized with the two numbers (m, j), and any quantity $\alpha(m, j)$ (e.g. the partition asymmetry) can be summed over the tree rooted at (M, j_F) as follows:

$$A(t_{J_F}) = (j_F + 1) \sum_{m=0}^{M-1} \nu^{M-m-1} \sum_{j=1}^{\nu-1} \alpha(m, j) + \sum_{j=1}^{j_F} \alpha(M, j). \quad (5)$$

The parameter M is the iteration order of the root node, and $j_F \equiv d(J_F)/\nu^M - 1$, where J_F is the root node.

If $\alpha(m, j)$ is the partition asymmetry of Eq.4, the sum Eq.5 can be simplified significantly and the Asymmetry

finally reads:

$$
\begin{aligned}
Q_T(t_{J_F}) = & \frac{1}{w(t_{J_F})}(1-\left(\nu^M-1\right)\mathrm{H}[j_f] \\
+ & \frac{1+j_f}{2}(-2+j_f+M(-1+\nu))\nu^M \\
- & \frac{(1+j_f)\mathrm{H}[\nu-1]}{(\nu-1)\nu}\left(\nu+(M(\nu-1)-\nu)\nu^M\right))
\end{aligned} \tag{6}
$$

with:

$$
\begin{aligned}
w(t_{J_F}) = & 1+\frac{\nu^M\left(-2+j_f+j_f^2\right)}{2} \\
+ & \frac{\nu^{M-1}M(1+j_f)\left(-2+\nu+\nu^2\right)}{2}
\end{aligned} \tag{7}
$$

and $H[n]$:

$$
H[J] = \sum_{k=1}^{J}\frac{1}{k}. \tag{8}
$$

In Fig. S1(c) we plot the Asymmetry Q_T as a function of the iterative order M for various self similar trees of the type shown in Fig. S1(b). The cases shown are $\nu = 3$ (green), $\nu = 5$ (red) and $\nu = 15$ (cyan). Note that j_F is set to 1, and in this case $d(t_{(1,M)}) = 2\nu^M$. The Asymmetry Q_T approaches quickly an M independent value that increases with ν. The dependency of Q_T with ν is shown in Fig. S1(d). Here we set $M = 10^3$, $j_F = 1$, and we can see that, as expected, Q_T increases monotonically with ν. Using the Q_T values, we can determine that the Bursersa and Protium architecture (main text, Fig. 5 and Fig. 6) are consistent with a self similar nesting model with $\nu \simeq 4$ and $\nu \simeq 6$ respectively.

2 Asymmetry weight functions

The Asymmetry values Q_T depend on the choice of weight function w_j. Provided that the choice of weight is consistent when comparing two or more graphs, the qualitative results from the Asymmetry metric are robust. In Fig. S2 we show the asymmetry values for three different weights.

The Asymmetry Q_T is calculated with the weight function used in the main text, shown in Eq. 2. The Asymmetry Q_4 is calculated over a weighted moving window, four nodes deep (the nodes included in calculating the Asymmetry Q_4 of the subtree are separated from the root of the subtree by at most four nodes). The weight function now is set to depend not only on the node j but also on the distance $h(j,n)$ of that node from the root

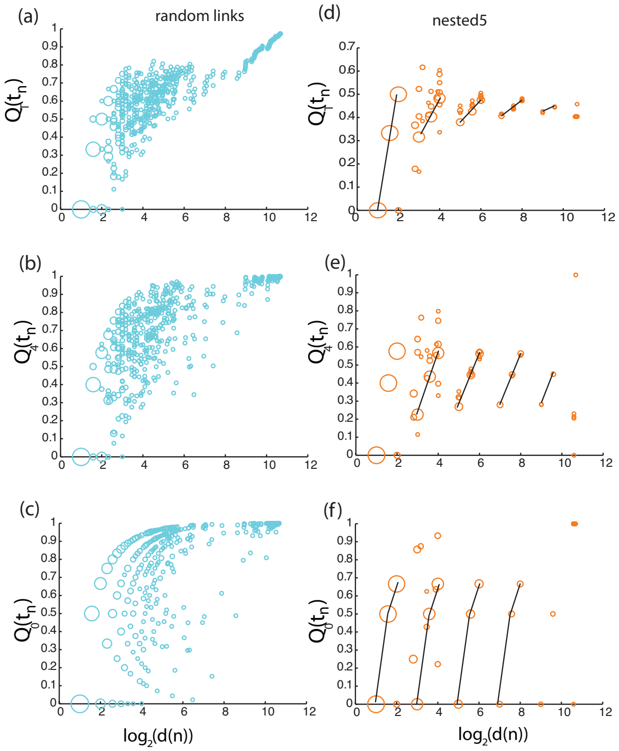

Figure 2: Asymmetry scatter plots of the random links and nested5 model. The size of the circles at each point (d, Q) indicates the number of nodes of the nesting tree with $d(n) = d$ and $Q(t_n) = Q$. (a),(b),(c): random links model, (d),(e),(f): nested 5. (a),(d): $w_j = w_j = d(j) - 1$, (b),(e): $w_j(n) = \frac{d(j)-1}{h(j,n)+1}\Theta(h(j,n) < 4)$, (c),(f): $w_j(n) = \delta_{j,n}$.

node n of the subtree:

$$
w_j(n) = \frac{d(j)-1}{h(j,n)+1}\Theta(h(j,n) < 4). \tag{9}
$$

$\Theta(x)$ is the step function, and imposes that the distance $h(j,n)$ cannot be larger than 4. This weight will produce a finite size averaging window, heavily weighted towards the root of the subtree.

Finally, Q_0 is calculated with a zero size averaging window, implemented with a Kroenecker delta weight function $w_j(n) = \delta_{j,n}$, so that:

$$
Q_0(t_n) = q(r_n, s_n). \tag{10}
$$

$Q_0(t_n)$ has zero memory and contains no information about the architecture of the two subtrees that join at node n.

The nesting trees that can be mapped to each other by successive reflections with respect to the vertical axes

passing through nodes are considered identical in our implementation. The number of non-homeomorphic binary trees of a certain degree d can in principle be enumerated (see Ref. [1] and references therein). As a result, the number of distinct Asymmetry values that map to a certain degree d is finite. For example, there are only two trees of root degree $d(n) = 4$.

Similarly, it can be trivially shown that

$$Q_0(t_n) = \frac{d(n) - 2j}{d(n) - j} \tag{11}$$

with $j \in \{1, ..., \lfloor j/2 \rfloor\}$, where $\lfloor p \rfloor$ is the integer smallest or equal to p. The Asymmetry values $Q_0(t_n)$ of the random links and nested5 models are shown in Fig. S2(c) and (f). For small $d(n)$, it is easy to enumerate the distinct Asymmetry values. The size of the data points in Fig. S2 is proportional to the number of vertices of the nesting trees that share the same order and Asymmetry $(d(n), Q_0(t_n))$. The distribution of $(d(n), Q_0(t_n))$ values carries information about the network architecture. For example, the periodicity of Fig. S2(f), highlighted with the black lines, is a signature of the self-similar nested model with $\nu = 4$, from which the nested5 model was created by random permutation of 5 lines. The data points with $2^2 < d(n) < 2^4$ that do not coincide with the black line are due to the five swapped lines in this realization of the random model.

The Asymmetry Q_4 (finite size averaging window) produces qualitatively similar results. In this case, each $Q_4(t_n)$ carries some information about the architecture of the subtrees that join at node n. The periodicity of Fig. S2(f) is still present in (e), albeit with a smaller amplitude. When the averaging window is of infinite size, as in Q_T in Fig. S2(d) the amplitude of the oscillations will asymptotically relax to a zero, and $Q_T(t_{n\to\infty})$ will be a constant that depends on the overall architecture.

These observations are reproduced when considering the average asymmetry $\bar{Q}_0(d)$ and $\bar{Q}_4(d)$, shown in Fig. S3(a) and (b).

3 Dual graphs and spanning trees.

In this section we discuss the connection between the nesting tree of the planar graph and the dual graph. The dual $G'$of a loopy planar graph G with no tree components (no tree subgraphs connected to G by a single edge) is a loopy planar graph with edges crossing the links of G and connecting adjacent loops (akin to the Delaunay triangulation of a Voronoi diagram). This graph has no tree components (no bridges) apart possibly from the outer edges connecting the peripheral loops to infinity (the outside of the finite graph). This graph is weighted, and we set the weight of each edge to be equal to the weight of G that it crosses.

The Economy tree $T(G')$ [2] of G' is a spanning tree of G' that is created as follows. The first edge of $T(G')$ is the edge E_1 with the minimum weight. The second edge, is the edge of $G'_1 = G' - E'_1$ (the remaining graph) having minimum value. For any subsequent edge, we similarly choose the minimum value edge of the remaining $G'_k = G'_{k-1} - E_k$, provided that it does not form a loop with

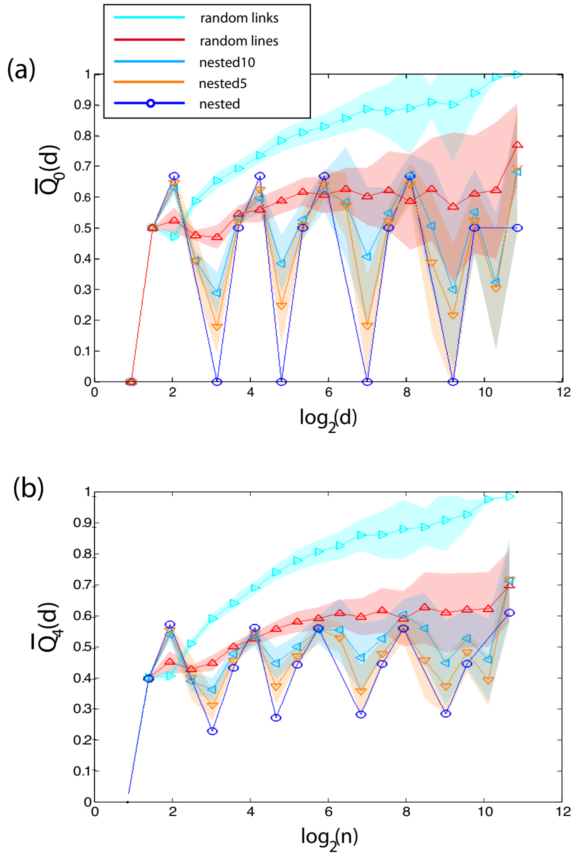

Figure 3: Average Asymmetry of computer generated graphs. The average Asymmetry is plotted as a function of the subtree degree $d(n)$. (a) $\bar{Q}_0(d)$ (no averaging over the node subtree in $Q_0(t_n)$) (b) $\bar{Q}_4(d)$: averaging four deep. Black line: nested model. Magenta: nested5. Green: nested10. Red: random lines. Blue: random edges. The colored area represents the standard error after averaging over 20 realizations of each model. When the asymmetry is not averaged over the whole subtree, the oscillations in the self-similar model are not damped. The results qualitatively follow those of Fig. 3(d) of the main text and Fig. 2.

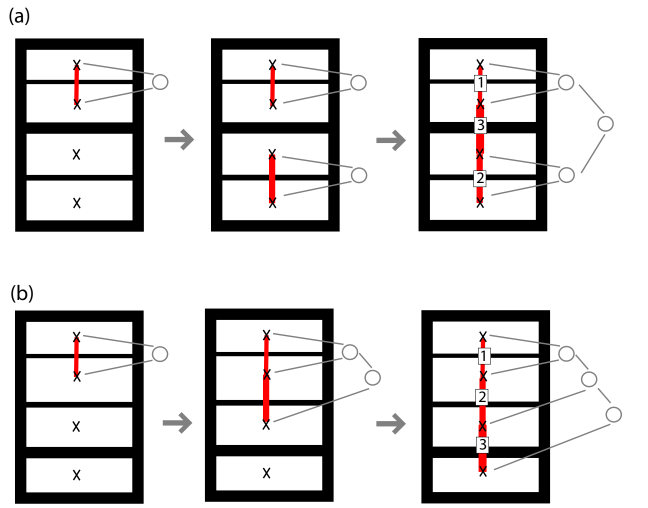

Figure 4: The tree representation can be projected on the dual graph of the original network. It will be a spanning tree on that graph. The thickness of the edge of the dual graph represents the thickness of the edge that separates the two real space loops.

the previously formed edges. Knowledge of the topology of $T(G')$ is not enough to characterize the architecture of G, as graphs with distinctly different architectures can share the same economy tree. An example of that can be seen in Fig. S4(b), where (if we ignore the connection to infinity) the two planar graphs shown have a linear economy tree $T(G')$.

The nesting tree $\tilde{G}$ is related to $T(G')$ as follows. When we choose the first edge of $T(G')$ E_1, we also create a new “lifted node” that connects the nodes of E_1. We repeat for all subsequent chosen edges of $T(G')$, as shown in Fig. S4. If they are contiguous to edges that have already been chosen, the new lifted node connects the lifted nodes of the contiguous edges rather than the actual nodes of the edges. This way, at every iteration of the process each connected part of the forming economy graph will be represented only by one lifted node. This node is the root node of that connected binary tree. The final tree that is produced when all nodes of G' have been connected by the economy tree is the nesting tree. Its projection onto the initial plane is the original economy tree.

References

[1] Toroczkai Z (2002) Topological classification of binary trees using the horton-strahler index. *Phys Rev E* 65:016130.

[2] Chartrand G (1985) *Introductory graph theory* (Dover).
